# The interactive work of narrative elicitation in person‐centred care: Analysis of phone conversations between health care professionals and patients with common mental disorders

**DOI:** 10.1111/hex.13440

**Published:** 2022-02-11

**Authors:** Matilda Cederberg, Andreas Fors, Lilas Ali, Anneli Goulding, Åsa Mäkitalo

**Affiliations:** ^1^ Institute of Health and Care Sciences, Sahlgrenska Academy University of Gothenburg Gothenburg Sweden; ^2^ Centre for Person‐Centred Care (GPCC) University of Gothenburg Gothenburg Sweden; ^3^ Region Västra Götaland, Research and Development Primary Health Care Gothenburg Sweden; ^4^ Psychiatric Department Sahlgrenska University Hospital Gothenburg Sweden; ^5^ Region Västra Götaland, Psychosis Clinic Sahlgrenska University Hospital Gothenburg Sweden; ^6^ Department of Education University of Oslo Oslo Norway; ^7^ Department of Education, Communication and Learning University of Gothenburg Gothenburg Sweden

**Keywords:** narratives, patient‐centred care, patient‐provider communication, person‐centred care, storytelling

## Abstract

**Introduction:**

Narratives play a central part in person‐centred care (PCC) as a communicative means of attending to patients' experiences. The present study sets out to explore what activities are performed and what challenges participants face in the interactive process of narrative elicitation, carried through in patient‐professional communication in a remote intervention based on PCC.

**Methods:**

Data were gathered from a randomized controlled trial (RCT) in a Swedish city where health care professionals (HCPs) conducted remote PCC for patients on sick leave due to common mental disorders. A sample of eleven audio‐recorded phone conversations between HCPs and patients enroled in the RCT were collected and subjected to conversation analysis.

**Results:**

Three interactive patterns in narrative elicitation were identified: Completed narrative sequences driven by the patient, question‐driven narrative sequences guided by the HCP, and narrative sequences driven as a collaborative project between the patient and the HCP. In the question‐driven narrative sequences, communication was problematic for both participants and they did not accomplish a narrative. In the other two patterns, narratives were accomplished but through various collaborative processes.

**Conclusion:**

This study provides insight into what challenges narrative elicitation may bring in the context of a remote PCC intervention and what interactive work patients and HCP need to engage in. Importantly, it also highlights tensions in the ethics of PCC and its operationalization, if the pursuit of a narrative is not properly balanced against the respect for patients' integrity and personal preferences. Our findings also show that narrative elicitation may represent an interactive process in PCC in which illness narratives are jointly produced, negotiated and transformed.

**Patient or Public Contribution:**

Stakeholders, including patient representatives, were involved in the design of the main study (the RCT). They have been involved in discussions on research questions and dissemination throughout the study period. They have not been involved in conducting the present study.

## 1 INTRODUCTION

During recent decades, attending to patients' narratives has been recognized as a way of making patients' experiences more central within health care contexts. Patients' illness narratives have been said to constitute the ‘voice of the lifeworld’ and to offer complementary perspectives to the biomedical ‘voice of medicine’.^1^, ^2^, ^3^, ^4^ During the same time, person‐centred care (PCC) has been widely recognized as an alternative to reductionist, disease‐centred biomedical models of care and adopted by many countries.^5^, ^6^, ^7^ In PCC, the core ontological assumption is that patients are first and foremost persons. This assumption serves as the guiding ethical principle in conducting health care.^8^, ^9^, ^10^ A closely related term is ‘patient‐centred care’, which is sometimes used interchangeably with PCC. In a recent review comparing the two concepts, the authors found more similarities than differences but concluded that the perspective of PCC was broader and more focused on the patient's whole life.^11^ Understanding the patient's experiences of their illness and its consequences for everyday life is a central aspect of conducting PCC and an important focus of patient‐professional interactions.^8^, ^12^, ^13^ When patients' experiences are given a prominent position in health care interactions, patients are likely to be more actively engaged and more satisfied with care.^14^, ^15^, ^16^ Previous studies on narrative elicitation in PCC have highlighted it as a complex interactive process requiring communicative skills, sensitivity and openness to an array of potential stories.^17^, ^18^ Health care professionals (HCPs) have described different communicative strategies they employ to encourage patients to narrate their experiences, for example, sitting down, having a calm demeanour, listening attentively and asking open‐ended and follow‐up questions.^16^, ^17^, ^19^, ^20^ However, incorporating aspects outside the medical realm into the health care encounter can put both patients and HCPs in a position of unease and uncertainty as to what is expected of them as narrators and listeners to lifeworld concerns.^18^, ^21^ Through interaction analyses, such as conversation analysis (CA), the interactive processes underpinning narrative elicitation in person‐centred communication, and what it requires of participants, could be further explored.

### 1.1 CA on narratives in health care interactions

CA is a method to describe patterns of naturally occurring interaction, which can be used to unpick patient‐professional communication. Within conversations, speakers are taking turns at talk. They are entitled to a single‐turn constructional unit (an utterance) and when that unit reaches a recognisable point of completion the speech initiative may alter between participants.^22^ Narratives and stories often stretch over longer sequences, consisting of multiple‐turn constructional units, which requires specific and subtle negotiations of speech initiative that may differ depending on whether the narrative is initiated by the speaker or solicited by the recipient.^22^ CA has been applied to study activities in health care communication directly or indirectly related to the production of patients' narratives.^23^, ^24^, ^25^, ^26^ Findings relevant for conducting PCC are, for example, how access to the first‐ and third‐turn slots authorizes HCPs to introduce topics and be in charge of the agenda,^27^, ^28^ and how patients nevertheless can find strategies to share their perspectives by going beyond the topical agenda.^24^ Patients' narratives are also shaped by the way HCPs pose questions: experience‐oriented, affective or general‐inquiry questions generally support narrative production,^23^, ^24^, ^29^ as does experience‐oriented formulations^24^ and tentative speech.^23^ Drew et al.^26^ and Kettunen et al.^23^ have also described the importance of leaving conversational space open in order for patients to contribute with their point of view, something which may be undermined if the conversation is too heavily standardized.^26^

However, with the exception of Drew et al.,^26^ none of these studies is conducted within the inferential framework of PCC, which constitutes a particular kind of institutional encounter by placing the patients' narrated experiences of their condition as the primary basis of care and treatment planning.^8^ Our starting point of this paper is that the normative appeal to practise PCC serves as a particular context for framing the interaction between HCPs and patients, and that narrative elicitation, especially when serving as a bannister for the realisation of the ethics of PCC in a microperspective, warrants further scrutiny and exploration. Our aim is to explore what activities are performed and what challenges participants face in the interactive process of narrative elicitation, carried through in patient‐professional communication as part of a remote PCC intervention.

## 2 METHODS

### 2.1 The intervention study

The present study is part of a larger randomized controlled trial (RCT) evaluating the effects of remote PCC as additional support, complementary to usual care, for patients on sick leave due to common mental disorders (CMDs).^30^ The RCT took place in an urban region in Sweden, and patients were recruited from nine primary health care centres. The intervention was administered remotely, through access to an interactive digital platform and through phone support with HCPs trained in PCC. Although the content of the interactive platform was adapted to suit the conditions of patients included in the study (mild to moderate CMD), the primary aim of the intervention was to create a partnership with the patient, based on their narratives, to support them in achieving what was identified as their health‐related goals. According to the evidence‐based PCC framework^8^, ^13^ used in the study, this is partly achieved through the narrative approach of the PCC phone conversations. The HCPs who worked with the intervention had varying degrees of experience in the theory and practice of PCC, and took part in regular meetings with experienced clinicians and researchers for continuous reflection and training in the ethical principles and practice of PCC. Part of this training consisted of reviewing some of each other's conversations and giving each other feedback on conducting PCC over the phone. None of the HCPs had any prior experience of working specifically with CMDs but participated in a half‐day lecture led by experts in CMDs. As the intervention was performed in addition to usual care, the patients were managed in parallel at their primary health care centre according to guideline‐directed care. When patients were allocated to the intervention, a phone conversation with the HCPs was scheduled to occur within a week or two. Unless the patient declined, these phone conversations were recorded, and these audio recordings are the material used for the present study.

### 2.2 Data collection

Data for the present study were collected between the start of the RCT in February 2018 and November 2018. During this period, three HCPs (two registered nurses [RNs] and one physiotherapist [PT]) worked with the RCT and a total of 22 initial phone conversations were recorded. Only initial conversations were included because they represented the first occasion for narrative elicitation. One recording was excluded due to sound disturbances. More than half of the remaining audio‐recorded conversations were conducted by one of the RNs (called RNa in the excerpts). To minimize the risk of a biased sample dominated by conversations led by the same HCP, we eliminated, at random, some of the recordings conducted by RNa. This resulted in a final sample of eleven audio‐recorded conversations, fairly evenly distributed between the three HCP (five recordings by RNa, three by RNb and three by the PT). All three HCPs were women, five of the patients were men and six were women. The length of the conversations varied from 23 to 62 min, and the 11 audio recordings amounted to a total of 462 min. Ethical approval was obtained from the Regional Ethical Review Board in Gothenburg, Sweden (DNr 497‐17, T023‐18 and T526‐18). Participants gave written consent to participation, and oral consent was given before starting recording at the beginning of each new conversation. The transcripts were treated with full confidentiality and names or identifiable characteristics have been altered.

### 2.3 Analytic procedure

The first author transcribed the conversations with attention to interaction details suitable for the present analysis, for example, emphasis, notations of pauses and micro‐pauses and overlapping speech (see the Supporting Information Appendix for transcript legend). The conversations were conducted in Swedish, and so was the transcription and analysis. Thereafter the analysis, as well as the excerpts from the transcripts, were translated into English. All recordings and their respective transcripts were examined by the first, second and last author, using CA.

The analysis was oriented to how narratives were achieved, how they were accomplished in the interaction and managed turn‐by‐turn.^22^, ^31^ First, narrative sequences in the conversations were identified and an initial grasp of their content and interactional form was sought. The narrative sequences were then discussed in terms of their location in relation to the overall phases of the conversations. Then, each phase across the 11 conversations was analysed separately to search for interactive patterns in how narratives were introduced, if they were elicited by the HCP or self‐initiated, how narrating occurred (or the lack thereof when sought) and what type of activity the narrative produced. Interactional patterns from all 11 conversations were then identified and analysed in terms of differences and similarities. Our analytical focus was thereafter directed to the opening sequences and the main project of the conversations. Excerpts including three different patterns were chosen to scrutinize the preliminary analysis in a data session with interaction scholars from other research fields. The results were then discussed among all coauthors, providing a further examination of the trustworthiness of the findings.

## 3 FINDINGS

### 3.1 Opening sequences

Overall, the conversations followed a general structure of an opening phase, followed by the main project of narrative elicitation, a summary phase and a closing phase. The opening phase included greetings, and in half of the conversations, there was a preparatory sequence explaining, for example, the aim of the conversation (its open‐endedness) and role expectations (patients choosing topics they find relevant). Excerpt 1 (Table 1) illustrates one such preparatory sequence (see transcript legend in the Supporting Information Appendix).

**Table 1:** Excerpt 1

1	HCP	Um and so with this being the first conversation today (.) um
2		Pernilla
3	Pat	Mm‐hm.
4	HCP	We talked a bit about that last time (.) kind of what it's about and so on (.) but
5		
6	Pat	Yes.
7	HCP	Um (.) actually it is it‐it's your choice what to like if‐if there's something in particular you want to talk about or address or something (.)'cause it's (1.5) you that (.) you know, you're supposed to um feel‐ yeah it should be about what you think is important to talk about (.) or what you want
8		
9		
10		
11		
12	Pat	Mm‐hm.
13	HCP	Um and then of course I'm in this conversation too so I'll (.) be asking questions and so on too but (.) uh just so that (.) yeah but you're the one running it, kind of, so it's like (.) it‐it's what suits you or what you feel if there's anything particular you want to take up (.) then (.) that should
14		
15		
16		
17		
18	Pat	Yes.

*Note*: HCP = health care professional, PT, woman. Pat = patient nr 11, woman.

In Excerpt 1, the HCP explains the intention of the conversation. In Line 7, the HCP begins her utterance with ‘actually’, framing what she is about to say as something that might be contrary to what is expected. She then goes on to explain that this conversation is supposed to be about matters that are meaningful to the patient, who can choose what to talk about. Next, the HCP clarifies how she will participate during the conversation, making it clear that the patient should be ‘running it’ (Line 15).

When a transfer from the opening phase to the main project occurred, this was explicitly signalled by the HCPs. Often, they used a ‘please go ahead’ or ‘where should we start,’ followed by an open‐ended question either directed retrospectively at what had happened, or to the patient's present status or circumstances. The HCPs could also suggest several possible topics for the patient to choose.

### 3.2 The main project: Shaping the conversations in narrative form

Narratives could occur by self‐initiation in any of the conversational phases, but their elicitation was at the core of the main project. Below, we will show patterns of how narrative elicitation was carried out in our material and the different kinds of interaction from which narratives emerged. There are patient‐driven narrative sequences, question‐driven narrative sequences and co‐constructed narrative sequences (Table 2). These patterns of interaction should not be understood as describing the entire conversations but only elicitation sequences within the conversations. One conversation could thus include sequences of patient‐driven narration, followed by a sequence of co‐construction, and so forth.

**Table 2:** Overview of interactive patterns and participation frameworks to achieve them

	Patient‐driven narrative sequences	Question‐driven narrative sequences	Co‐constructed narrative sequences
**Narrative characteristics**	‘Story‐like’ narratives, without gaps or uncertainties. The narrative follows a time line and contains descriptions of inner and outer events	Fragmented accounts providing marginal insight into the patient's experiences. They lack one or several narrative qualities	Narratives have ‘story‐like’ qualities but are unresolved in terms of content or meaning
**Participation framework**	The patient takes the floor and tells a narrative over several turns. The HCP lets the patient have the floor and gives interactional support through minimal responses	The patient does not take the floor to tell a narrative and struggles with the HCP's requests. Lack of any extended turns at talk	The patient narrates experiences but gaps, troubles or questions invite the HCP to take a more active part in accomplishing the narrative, by asking questions or sharing interpretations

Abbreviation: HCP, health care professional.

### 3.3 Patient‐driven narrative sequences

Patient‐driven narrative sequences are sequences in the conversations where the patient held the initiative in telling a narrative. The narratives produced are ‘story‐like’ in terms of giving insight into a line of inner and outer events. The excerpt below (Table 3) is an example of such a sequence. Here, the HCP hands the floor over to the patient and thereafter holds a nondirective role. While the patient tells her story, the HCP remains in the background, demonstrating her ongoing attention by frequent mhm's, but not interfering with the patient's narration (see transcript legend in the Supporting Information Appendix).

**Table 3:** Excerpt 2

1	HCP	Alright then (.) please go ahead, tell me how are you doing?
2	Pat	Um (.) you mean right now or during the time I've been‐‐?
34	HCP	Well you can tell me how you're doing right now, and how you felt when it happened, how it happened, or (.) yeah.
5	Pat	((Clears her throat))
6	HCP	Mm‐hm.
7	Pat	Uh okay I'll start (.) from the beginning then I guess.
8	HCP	Mm‐hm.
9	Pat	So it was two months ago (.) more or less
10	HCP	Mm‐hm.
11	Pat	That (.) I just felt I couldn't take it anymore
12	HCP	Mm‐hm.
13	Pat	Um (.) I uh (.)I cried I was tired
14	HCP	Mm‐hm.
15	Pat	Uh my (.) friend had just passed away
16	HCP	Mm‐hm.
17	Pat	And of course that just made it all so much harder
18	HCP	Mm‐hm.
19	Pat	I felt I couldn't concentrate any longer I started having memory lapses, I started (.)
20		
21	HCP	Mm‐hm.
22	Pat	Uh feeling like everything was difficult, I was getting more and more like (.) well depression[like]
23		
24	HCP	[Mm]
25	Pat	I couldn't see anything bright there was only this (.) complete darkness all around me
26		
27	HCP	Mm‐hm.
28	Pat	And then I decided that I couldn't (.) I couldn't keep on working any longer
29		
		Omitted 19 turns
30	Pat	So I've been on sick leave now for (.) two months. Uh (.) yesterday was my first day back at work
31		
32	HCP	[Mm‐hm.]
33	Pat	[and] I'll be working twenty‐five percent of full‐time uh
34	HCP	Mm‐hm.
35	Pat	(.) until the start of my summer holiday (.) in a month from now
36	HCP	You started working today, you said?

*Note*: HCP = health care professional, reg. nurse (a), woman. Pat = patient nr 6, woman.

In Excerpt 2 (Table 3), the HCP solicits a narrative by introducing possible topics for the patient as a starting point (Line 3). The patient accepts the invitation to narrate (Line 7), sets the frame for her story by stating that she will ‘start at the beginning’, and hints at the entrance point for her storyline (Line 9). She then tells her story in a chronological fashion until returning to the present time (Line 30), indicating the completion of this particular narrative sequence. Through the narrative of how it all started a couple of months ago, the patient describes how she experienced symptoms (Lines 13, 19, 22) and the events linked to her deteriorating psychological condition (line 15+ in the omitted turns). In doing so she also produces an account that makes her current sick leave understandable, and justifiable. The narrative, which is delivered without any gaps in the interaction flow, comes across as complete—a story acknowledged by the HCP without any requests for clarification or elaboration. Every HCP turn throughout the sequence is used for listener tokens, allowing the patient to tell her story without interrupting. When the patient wraps up, by returning to present time circumstances, the HCP aligns with the previous turn (Line 35), by directing her question towards the present rather than to earlier, uncommented, parts of the sequence.

### 3.4 Question‐driven narrative sequences

In question‐driven narrative sequences, the project of narrative elicitation is not carried out smoothly. The participants seem to be struggling with the contributions of the other and they lack any extended turns of talk. The excerpt below (Table 4) illustrates one such sequence in the material. Overall, this was the least frequent pattern of narrative elicitation efforts. Other similar sequences were shorter and typically resolved sooner.

The sequence in Excerpt 3 illustrates the first minutes of the conversation and is only preceded by a short sequence of greetings. Throughout the sequence, interaction is problematic. The professional makes several attempts to shift the speaker initiative from herself to the patient, but these are not successful. The HCP's first question, ‘How are you doing’, is an open, neutral, quotidian question, but there is an element of ambiguity as it can be understood either as a common greeting or, given the health care context, as a request for information. The ambiguity of the previous turn remains as the patient hesitates (indicated in the excerpt by the micropauses) and gives a vague and minimum reply to how he is feeling this very day. The follow up from the HCP, a request for the patient to elaborate on his answer by describing what ‘pretty good’ is like (Line 3), receives a dispreferred response.^27^ The patient hesitates multiple times, exhales loudly, and struggles to answer the request (Lines 4–11). Already in Line 11, he wraps up his answer by summarizing his status (overall I feel pretty good) followed by a problem formulation (I've been sleeping very poorly so I'm tired), which could be read as an account of his lack of elaboration, or alternatively, an initiative to change the topic. In her following turn, the HCP seeks clarification on the extent of the sleeping problem (you mean last night) and the patient's short, confirmative reply closes the topic. On Line 17, the HCP instead returns to the previous topic of the patient's current status and requests an elaboration by suggesting a comparison of how he is doing now with how he has been doing earlier. The patient again acknowledges the request in the previous turn, but in a minimally informative way. This pattern continues throughout the sequence, giving the overall impression that the patient understands the HCP's requests, and his minimalistic, but accurate, replies can be understood as a compromise not exposing more of himself than necessary. Respecting another person's integrity thus becomes a delicate matter when pursuing to understand more about *a person* who is restrictive in the information they may want to give as *a patient*. In sum, the question‐driven narrative sequences are characterized by the patient not taking on the role of narrator and repeatedly handing the speech initiative back to the HCP. In contrast to the patient‐driven narrative sequences, the question‐driven sequences require more coaxing from the HCP. Still, the interaction takes on a more conventional question–response pattern.

**Table 4:** Excerpt 3

1	HCP	How are you doing?
2	Pat	(.) Uh today uhm (.) I'm doing pretty (.) good today, I'd say(.) eh‐
3	HCP	Mm‐hm (.) Can you describe a bit what pretty good is like?
4	Pat	(3.8) ((Exhales loudly)) I (.)feel (2.3) I feel uh (.) optimistic
5		
6	HCP	Mm‐hm.
7	Pat	Uh (1.3) I feel uh (.) sort of happy
8	HCP	Mm‐hm.
9	Pat	Uh (2.8) I feel like I (.) contribute something
10	HCP	Mm‐hm.
11	Pat	Uh (3.1) yeah overall I feel pretty good. I've been (.) sleeping very poorly so I'm tired but uh
12		
13	HCP	Mm‐hm you mean last night?
14	Pat	Yeah last night
15	HCP	Mm‐hm.
16	Pat	Exactly.
17	HCP	Uh‐huh (2.7) Okay, um (.) so you (.) if you were to compare this with the recent past would you say you are doing much better now then?
18		
19		
20	Pat	(.)Yeah um (.) well it (.) varies a bit but
21	HCP	Mm‐hm.
22	Pat	overall it's sort of gotten better.
23	HCP	Mm‐hm that's nice to hear mhm (.) uh (.) do you know what has made you feel better or have you done something particular yourself?
24		
25		
26	Pat	Uhh I've started ^3^ I've started (.) I don't quite know how to say but I've started trying to value life a bit more
27		
28	HCP	Mm‐hm.
29	Pat	Then I've also been in contact with my psychiatrist, we've (.) made some changes in the dosage of my (.) my medications
30		
31	HCP	Mm
32	Pat	which I think has (.) helped a bit
33	HCP	Mm‐hm (3.1) and so you feel like it's working better now?
34	Pat	Yeah started working out as well (.) I think that also
35	HCP	Mm‐hm.
36	Pat	helps a bit.
37	HCP	Yes definitely (.) mm‐hm (.) very good. Uh (2.1) if you were to describe how you're doing right now (.) um (3.2) what would you say, how‐‐?
38		
39		
40	Pat	(3.7) Well like I said it can be very different from one day to the next, but
41		
42	HCP	Mm‐hm.
43	Pat	um (.) a good day is like any normal day like, happy, optimistic, creative uh
44		

*Note*: HCP = health care professional, reg. nurse (a), woman. Pat = patient nr 1, man.

### 3.5 Co‐constructed narrative sequences

In co‐constructed narrative sequences, the narrative is treated as more of a collaborative project. This pattern will be illustrated by three excerpts from two different conversations. The first two excerpts display the joint elaborations of a narrative displaying gaps and the third excerpt shows how a narrative becomes a means for a joint reflective process.

### 3.6 Jointly elaborating the content of a narrative

In the excerpt below (Table 5), the HCP elicits a narrative around the circumstances preceding the patient's sick leave. Through her narrative, the patient discloses that this is an emotionally challenging subject.

In Excerpt 4, the HCP elicits a narrative about what happened that caused the patient to take sick leave (Lines 1–3). She starts to describe the situation at her current job, explaining some related circumstances that contributed to her reaching a tipping point (Lines 4–24). Throughout these turns, her frequent use of epistemic markers such as ‘probably’ and ‘I guess’ reveals uncertainty as to what happened.^32^ In Line 24, she stops her sentence short, as she starts crying. The narrative the patient begins telling in this sequence conveys a vague story of failure, which is charged with emotions, and throughout this sequence, the HCP does not intervene much. The most prominent participation from the HCP is her frequent use of listener tokens (mm‐hm), signalling that she is paying attention and that the patient may continue speaking. In Lines 44–45, after an utterance that could be interpreted as a story exit (Line 43), she requests clarification of the conditions of the patient's employment. The patient responds by further explaining the circumstances of her becoming ill, thus staying in the narrative a bit longer. Shortly thereafter, they change the topic. After about 5 min, the HCP returns to the subject, as manifested in the excerpt below (Table 6).

In Excerpt 5, the HCP returns to the topic of what caused the patient's sick leave, as was initiated in the previous excerpt (Excerpt 4, Table 5). Throughout the sequence, the HCP poses several questions directed both at the factual circumstances at work (Lines 1–2, 24, 26–27) and at the patient's understanding of what happened (Lines 18–19, 29). Here the HCP does not use open‐ended questions, which are traditionally seen as the ideal practice for encouraging narrating.^29^ Her questions are instead delivered in the form of assertions, which directs the patient's subsequent turn and prompts her to continue by accepting or declining the embedded suggestion. The first suggestion, in Line 1, is followed by a confirmation, in which the patient elaborates in more detail on how the stress at work affected her. Refraining from the open‐question format produces another kind of relation to the narrative, one in which the previous content is challenged (Line 1–2 ‘but did you feel stressed’, Line 24 ‘but you didn't get any support’, Lines 26–27 ‘but did you ever talk to your employer’), until alignment in what happened is reached (Lines 34–39). In the patient's subsequent turn, the previously noticed uncertainty (Excerpt 4) towards what happened is no longer evident, as she either aligns (e.g., Line 20–23) or not (Line 28) with the HCP's suggestions. Although the HCP provides suggestions, it is the patient who, in her turns and in her own words, identifies the conditions that made her situation unbearable (Lines 20 and 22). Towards the end of the sequence, the HCP reformulates her understanding of what the patient has told her, acknowledging the story's credibility and validity, and by using the pronoun ‘you’ (in the sense of a general, impersonal pronoun), she normalizes what happened to the patient as something which is relatable and understandable also in a general sense. Throughout this excerpt, the story of what happened becomes clearer, challenging the uncertainty and personal failure‐tendency visible in the patient's initial account.

**Table 5:** Excerpt 4

1	HCP	But uh how (.) how uh what happened or could you tell me something about what happened, or how you ended up on sick leave?
2		
3		
4	Pat	Um. Well I guess it's been really stressful at work
5	HCP	[Mm‐hm.]
6	Pat	[I'm] quite new at my job
7	HCP	Mm‐hm.
8	Pat	I've been at this workplace for a few years
9	HCP	Mm‐hm.
10	Pat	(.)And then uh last year
11	HCP	Mm‐hm.
12	Pat	in 2017, I started in a new position
13	HCP	Mm‐hm.
14	Pat	((exhales)) what I'm trained for
15	HCP	Mm‐hm.
16	Pat	Realt‐ real estate agent
17	HCP	Mm‐hm.
18	Pat	Uhh and it was a ((sighs)) I got a junior position so it was a new position at the company as well
19		
20	HCP	Mm‐hm.
21	Pat	Um (.) I suppose it was just too much
22	HCP	Mm‐hm.
23	Pat	that I didn't get any training (.) which I was supposed to get ((starts crying))
24		
25	HCP	Mm‐hm.
26	Pat	I'm sorry, it's kind of hard
27	HCP	No, no, I'm sorry if I've‐‐
28	Pat	Yes.
29	HCP	Mm‐hm.
30	Pat	It's alright (.) It's good to talk about it
31	HCP	Mm‐hm.
32	Pat	((crying)) But so I guess you could say it got to be too much
33	HCP	Mm‐hm.
34	Pat	So I didn't get the help ((inhales)) the support I was supposed to get
35	HCP	Mm‐hm.
36	Pat	So I guess you could say it had been like that since last summer
37		
38	HCP	Mm‐hm.
39	Pat	but I didn't really realize it myself, it was actually a (.) one of my co‐workers who asked me ‘how are you doing really?’
40		
41	HCP	Mm‐hm.
42	Pat	And then I realized that (.) I wasn't doing so great
43	HCP	Mm‐hm (.) So are you alone at work or uh do you share duties with anyone or, how uh‐‐?
44		
45	Pat	Yeah no we're, I have some colleagues (.) who also have (.) who do the same things
46		
47	HCP	Mm‐hm.
48	Pat	They're just much more experienced than I am
49	HCP	Mm‐hm.
50	Pat	So they (.) I haven't managed to get a grip on how I ought to organize it
51		
52	HCP	Mm‐hm.
		13 turns omitted in which the patient gives further examples of her struggles to manage the stress at work.

*Note*: HCP = health care professional, reg. nurse (a), woman. Pat = patient nr 4, woman.

**Table 6:** Excerpt 5

1	HCP	Um (.) but (.) did you feel stressed while working (.) at your new job then? Was it the stress that caused you to (.) or?
2		
3	Pat	Yes. Yeah that was it, it felt like I (.) You never unwind, I was always bringing the stress of the job home with me
4		
5	HCP	Mm‐hm.
6	Pat	Like I'd be checking my work email when not at work
7	HCP	Mm‐hm.
8	Pat	And (.) well I didn't answer the phone though (.) I did draw the line there
9		
10	HCP	Mm‐hm.
11	Pat	But (.) if something occurred to me just as I was about to go to sleep I'd have to email it to myself just so I wouldn't forget it and so that I could (.) feel able to go to sleep properly
12		
13		
14	HCP	Mm‐hm.
15	Pat	and not (1.3) so I could let go of whatever it was
16	HCP	Mm‐hm.
17	Pat	There was a lot of stuff like that
18	HCP	Mm‐hm. So (1.3) so you know what it was that caused you to end up there?
19		
20	Pat	Yes it was all too much (.) too much new stuff for me
21	HCP	Mm‐hm.
22	Pat	Lack of knowledge. Which I didn't know how to handle (.) uh, it was
23		
24	HCP	But you didn't get any support‐‐?
25	Pat	Not enough (.) no I didn't.
26	HCP	Mm‐hm. But (.) did you ever talk to your employer or your boss or did you try to let anyone know how you were feeling before, or?
27		
28	Pat	No. [Not enough.]
29	HCP	[You didn't really notice] it yourself then?
30	Pat	No (.)'cause I don't think I understood really (.) I thought I knew my own body
31		
32	HCP	Mm‐hm.
33	Pat	so to speak, but it
34	HCP	Mm‐hm, yeah it's not always self‐evident and also when you're new you want (.) to perform as well as possible
35		
36	Pat	Mm‐hm.
37	HCP	Show that you're capable and that you can manage (.) but unfortunately sometimes it's too much
38		
39	Pat	Yes, that's the way it was

*Note*: HCP = health care professional, reg. nurse (a), woman. Pat = patient nr 4, woman.

### 3.7 The narrative as a means for joint reflections

Excerpt 6, below (Table 7), is taken from another conversation and presents a slightly different example of a co‐constructed narrative also concerning a retrospective understanding of what happened before the sick leave. The excerpt begins in the middle of a longer narrative in which the patient reflects upon different aspects of his life and their potential connection to his recent illness and related struggles.

In Excerpt 6, the patient narrates the circumstances preceding his sick leave to the HCP. At the beginning of the sequence, he reflects on the topic of why he got a depression and when it started, while  also describing a process of change he is currently in the midst of. In Lines 12–16, he begins introducing present tense verbs, signalling the potential wrapping of this particular narrative. The hints on an exit are reinforced in Lines 18–20, with his summarizing statement that it feels good getting help sorting this out. In Lines 21–22 there is a brief moment of overlapping speech, but by switching to giving listener support (Line 23), the HCP lets the patient continue his turn, and he gives a clearer story exit in Line 25. Thereafter, a change in speakership occurs, and through the rest of the sequence, it is the HCP who delivers the extended turns and gives a tentative interpretation of what the patient has described happening. She explicitly positions her interpretation as tentative by referring to her being the second‐hand source of experience (Line 32–33, ‘from what you've told me’), and continues on to use this interpretation as a base for supporting the solution the patient has found to problems in his current situation (Lines 34–42). Beginning in Line 46, by referring to the patient's process as ‘a journey’, and in Line 48–49, by suggesting that what he describes is a turning‐point, she addresses aspects of the patient's overall illness narrative and contributes to his narrative understanding, that is, his meaning‐making process. In Line 51, she signals that her reformulation is finished, and explicitly requests the patient's perspective on what she has suggested. He confirms her contribution and continues to elaborate on the topic (outside excerpt).

In contrast to a patient‐driven narrative, this narrative is not told as a completed story with its morals already sorted out. Rather, both participants relate to the narrative with reflections on it. Co‐constructed narratives have this participatory evolution of the story in common, although, as we have shown, the incentives can differ. In Excerpts 4 and 5 (Tables 5 and 6), the narrative of what caused the sick leave evokes emotional reactions and, initially, a general sweeping chain of events. Lingering on the topic, and returning to it later on in the conversation, shows an initiative from the HCP to get the patient to elaborate on the background story. In Excerpt 6 (Table 7), this elaboration is driven mainly by the patient's own reflective stance towards what happened, and the HCP participates in the reflective process with tentative interpretations.

**Table 7:** Excerpt 6

1	Pat	And then at work this past fall when I came back from parental leave I ha‐(.)ven't felt comfortable and I've wanted to come ba‐(.)ck to the city, and I've (1.2) completely (1.7) how should I put it (.) I've struggled to find meaning in, in work and with getting things done, and I've always been pretty efficient and (1.9) done a lot and helped and haven't had any problem with working uh evenings and weekends and, putting in extra hours and all that and (.) then all of a sudden I wasn't that person anymore and things started not going so well and I, I felt (2.6)yeah I never really felt alert and happy
2		
3		
4		
5		
6		
7		
8		
9		
10		
11	HCP	No
12	Pat	Not at home either so so I realize it's been building (.) It's been (.) under way I can see that now
13		
14	HCP	Mm‐hm.
15	Pat	And also that I know (.) and I've known it before a lot throughout my life, things I've been (.) problems I could never really get a grip on or (.) deal with so it's (1.3) now I've gotten more help with that too and
16		
17		
18		
19	HCP	Yes
20	Pat	understand a bit more how I (1.4) am, because that also feels good
21	HCP	Yeah, really good [yes, for sure that's]
22	Pat	[Even if it is]
23	HCP	Mm‐hm.
24	Pat	a lot to (.) work through ((laughter))
25	HCP	Yeah I can understand that and I'm sure you've talked to your psychologist about this but uh for me as I'm listening to you now, it sounds like (.) yeah, when you became a parent (.) when you got your family and all, life took a new turn and your old life and the way you used to work (.) it was no longer what was really good for you?
26		
27		
28		
29		
30		
31	Pat	No.
32	HCP	But this feels like (.) I don't know for sure but from what you have told me it sounds like this new job was (.) maybe (.)a much better job in the situation you're in now
33		
34		
35	Pat	Mm‐hm.
36	HCP	The way [your life is now]
37	Pat	[Yes, exactly]
38	HCP	now it's maybe (.) more your family that you would like to prioritize
39		
40	Pat	Mm‐hm.
41	HCP	You talk a lot about your daughter and how she must (.) yeah, you seem tremendously devoted to your daughter of course
42		
43	Pat	Mm‐hm.
44	HCP	like all dads or parents are so (.) that (.) yeah, it feels very (1.5) it feels clear to me when you talk about it, like about your journey here now
45		
46		
47	Pat	[Mm‐hm.]
48	HCP	[that] there was a turning‐point that uh (.) that life went in a different direction
49		
50	Pat	For sure
51	HCP	Could this be how it is? Does it feel this way to you?
52	Pat	(1.3) Yeah (.) yeah it is like that and then (.) you never really (.) have time to understand it [I feel]
53		
54	HCP	[No.]

*Note*: HCP = health care professional, reg. nurse (b), woman. Pat = patient nr 5, man.

## 4 DISCUSSION

In the present study, CA was used to explore the activities and challenges of narrative elicitation in PCC. We identified three distinct patterns of interaction that follow the HCPs elicitation of a narrative: patient‐driven narrative sequences, question‐driven narrative sequences and co‐constructed narrative sequences. By analysing PCC interactions in situ, our findings extend previous knowledge and add insight into how the work of PCC is carried out in communication between HCPs and patients. When HCPs seek to practise PCC and use narrative elicitation to gain insight into the experiences of their patients, it is not a straightforward task. As others have pointed out, it requires both sensitivity and communicative skills.^17^ Our findings bring forth that it also requires communicative flexibility in adapting to the different narrative styles that patients and interactions will expose. This is most evident in the question‐driven narrative sequences in which the pursuit of a patient's narrative must be balanced against the respect for patients' integrity and personal preferences. This also becomes a question of not letting routines get in the way of the ethics they are intended to enable, such as understanding the resources and preferences of the person.^8^

Several other aspects can be important in understanding why some patients may struggle with telling narratives. First, telling illness narratives requires complex communicative abilities. Articulating the experiences related to an illness demands both a capacity to recall the experience and the ability to transform that experience into an account of a sequenced chain of events and actions recognisable to someone who was not present at the time they occurred. Furthermore, rather than brief answers, narrative elicitation requests extended stories, which patients may be unfamiliar or uncomfortable with.^21^ However, in our material, this did not occur to any great extent. On the contrary, although question‐driven narrative sequences did occur in some of the conversations, they were in clear minority to the other two patterns.

We suggest that to facilitate for patients and HCPs to engage in PCC communication encompassing a narrative structure, the concept of framing is of relevance.^33^ Explicitly framing the conversation as concerning the patients' experiences of their illness, could enable a shared understanding of the agenda and potentially the orientation towards the narrative as a shared project. It also opens up the possibility of renegotiating, in case this agenda does not sit well with what the patient expects or wants. The social norms of a health care interaction in line with PCC may not be the preferred interaction style of all patients or HCPs, but by explicating the expectations, chances that misunderstandings will distort the interaction could possibly be minimized.

It is also possible that the circumstances of the present study, where the conversations took place over the phone between participants who had never met before, impacted participants' willingness and ability to narrate. Research on health care conversations by phone indicates that patients' openness, self‐exploration and disclosure are not as dependent on the communication mode (phone or face‐to‐face) as on other personal or interpersonal factors; some find it helpful talking about personal matters by phone, others do not.^34^ However, the phone setting obstructs most means of physical communication, something that may limit the repertoire of strategies HCPs otherwise can use, to encourage patients' narratives.^17^, ^23^ However, lacking visual cues can increase the significance of the voice as a medium. HCPs practising PCC via phone described how listening attentively to a patient meant not only reflecting on the wording of what they expressed but also paying attention to their tone of voice and expressions of mood, and allowing silences to occur.^19^

Finally, regarding the other two patterns of patient‐driven narrative sequences and co‐constructive narrative sequences, we reflect on the former as a rather classical illness account owned by the patient in terms of both content and moral, a story narrated by the patient with the HCP giving listener support. The co‐constructed narrative sequences display a collaborative stance towards understanding the expressions of illness and their meaning in the patient's life.^1^ Consequently, we find that the activity of narrative elicitation in person‐centred communication has potential beyond accessing the patient's experiences; it may also serve as a transformative process in which the understanding of illness is jointly renegotiated.

The findings in this article derive from conversations with patients with CMDs conducted by phone. This raises the question as to how relevant the findings are for conversations taking place in face‐to‐face settings, and for other conditions than CMDs. Rather than make generalisable claims, our findings should be seen as adding to the body of knowledge highlighting PCC as an interactional practice requiring flexibility and adaptation to the patient's communicative preferences, as well as to the specific health care context. Another limitation is the small number of eleven conversations distributed on three HCPs included in our sample. A larger corpus of conversations, with a greater variety of HCPs, would perhaps disclose patterns of communication that were not represented in our sample.

## 5 CONCLUSION

This study provides insight into what challenges exist and what can be accomplished through narrative elicitation in the context of a remote PCC intervention. Importantly, it highlights tensions inherent in the ethics of PCC and its operationalisation, if the pursuit of a narrative is not properly balanced against the respect for patients' integrity and personal preferences. This provides another example of the importance of HCPs communicative flexibility and sensitivity in practising PCC, and we do not find it unlikely that this scenario resonates with the experiences of some patients and HCPs. Practising PCC is not a linear process, the patient's narrative does not always begin a health care process and pave the way for partnerships. Sometimes, the patient narratives could obstruct this process, and another way forward must be found. Furthermore, the patient's narrative is sometimes understood as a singular account, delivered by the patient on one particular occasion. In these conversations, narratives were delivered, returned to and elaborated, throughout the entirety of the conversations. Our findings also show that narrative elicitation may represent an interactive process in PCC in which illness narratives are jointly produced, negotiated and transformed.

## CONFLICT OF INTEREST

The authors declare that there is no conflict of interest.

## AUTHOR CONTRIBUTIONS

The first, second and last authors conceptualized the study and conducted the initial analyses. The first author collected and transcribed the data and wrote the first draft. All authors participated in validating the analysis and reviewing the final draft.

## Supporting information

Supporting information

## Data Availability

The data that support the findings of this study are available upon reasonable request from the corresponding author, Matilda Cederberg. The data are not publicly available due to privacy and ethical reasons.
