# Supplementary material for: The interactive work of narrative elicitation in person‐centred care: Analysis of phone conversations between health care professionals and patients with common mental disorders
Source: Health Expect. 2022 Feb 11;25(3):971–83. doi: 10.1111/hex.13440 (PMC9122427; doi:10.1111/hex.13440)
Supplement: Supplementary file 1 — Supporting information [file HEX-25--s001.docx]

# Appendix

Transcript legend

[ beginning of overlapping utterance

] end of overlapping utterance

(.) micro pause

(1.3) pause lasting 1.3 seconds

(xx) inaudible

- cut off sound

- - cut off utterance

- - ? cut off utterance with intonation of a question

Underlining emphasis or stress

, continuing intonation

? intonation of a question

. falling intonation

(( )) Transcriber’s description
